# Optimizing Hydrogen Production in the Co-Gasification Process: Comparison of Explainable Regression Models Using Shapley Additive Explanations

**DOI:** 10.3390/e27010083

**Published:** 2025-01-17

**Authors:** Thavavel Vaiyapuri

**Affiliations:** College of Computer Engineering and Sciences, Prince Sattam bin Abdulaziz University, Al-Kharj 11942, Saudi Arabia; t.thangam@psau.edu.sa

**Keywords:** thermochemical conversion, biomass gasification, clean energy, explainable artificial intelligence, Shapley Additive Explanations framework, summary plot, force plot

## Abstract

The co-gasification of biomass and plastic waste offers a promising solution for producing hydrogen-rich syngas, addressing the rising demand for cleaner energy. However, optimizing this complex process to maximize hydrogen yield remains challenging, particularly when balancing diverse feedstocks and improving process efficiency. While machine learning (ML) has shown significant potential in simulating and optimizing such processes, there is no clear consensus on the most effective regression models for co-gasification, especially with limited experimental data. Additionally, the interpretability of these models is a key concern. This study aims to bridge these gaps through two primary objectives: (1) modeling the co-gasification process using seven different ML algorithms, and (2) developing a framework for evaluating model interpretability, ultimately identifying the most suitable model for process optimization. A comprehensive set of experiments was conducted across three key dimensions, generalization ability, predictive accuracy, and interpretability, to thoroughly assess the models. Support Vector Regression (SVR) exhibited superior performance, achieving the highest coefficient of determination ($R^{2}$) of 0.86. SVR outperformed other models in capturing non-linear dependencies and demonstrated effective overfitting mitigation. This study further highlights the limitations of other ML models, emphasizing the importance of regularization and hyperparameter tuning in improving model stability. By integrating Shapley Additive Explanations (SHAP) into model evaluation, this work is the first to provide detailed insights into feature importance and demonstrate the operational feasibility of ML models for industrial-scale hydrogen production in the co-gasification process. The findings contribute to the development of a robust framework for optimizing co-gasification, supporting the advancement of sustainable energy technologies and the reduction of greenhouse gas (GHG) emissions.

## 1. Introduction

Rapid industrialization and population growth have resulted in a significant increase in energy demand. A significant portion of global energy consumption relies on fossil fuels, which may potentially raise the GHG emissions, particularly carbon dioxide (CO_2_) [1]. The far-reaching consequences of this trend, with detrimental effects on the ecosystem, have alarmed the world leaders and policymakers to move towards an environmentally benign energy source. Parallel to this, worldwide plastic production has increased steadily in order to satisfy the demands of the international market. Plastics, being composed of petroleum-based materials, have contributed to the depletion of non-renewable fossil fuels as a consequence of this phenomenon. Beyond that, the accumulation of plastic waste without a proper disposal system endangers the health of humans and animals by contaminating groundwater [2]. This has necessitated urgent research for a sustainable treatment of plastic waste. Numerous approaches have been utilized to alleviate this issue, but recycling plastic waste into fuels rich in energy has been recognized as the most favorable strategy to address challenges faced globally and move towards a more sustainable future [3]. Considering the rapid growth in energy consumption and the pressing environmental issues, the pursuit of clean energy production is a vitally important research endeavor. Hydrogen is widely acknowledged as a potential clean energy carrier due to its applicability and versatility. Moreover, hydrogen production can transform transportation by powering hydrogen fuel cells or by being converted into liquid fuels. It provides a clean alternative to fossil fuels, reducing carbon emissions while maintaining energy efficiency and advancing the transition toward sustainable mobility. The rapid development of hydrogen technology and growing energy demand has driven many countries to prioritize hydrogen development in their national strategies and implement measures to meet their sustainable development goals [4]. As hydrogen is globally recognized as an important energy carrier in international decarbonization strategies, there is rising attentiveness toward establishing the sustainability of hydrogen production. The plastic wastes containing hydrocarbons are considered as an excellent feed for hydrogen production with minimal GHG emissions [5]. The majority of nations are therefore eager to promote innovations in clean hydrogen production from plastic wastes and to boost their economy [6].

Thermochemical methods have recently received increased attention from the scientific community, governments, and industry as a promising versatile platform for producing hydrogen-rich gases from plastic wastes due to their high conversion efficiency and high process yield [7]. Amongst all diverse thermochemical methods, gasification has stood out as a key technology to provide a significant framework for large-scale conversion of plastic wastes with reduced GHG emissions. However, gasification can face challenges such as tar formation, high energy requirements, and incomplete conversion of feedstocks. Co-gasification, which combines plastic waste with other materials like biomass, offers several benefits, including improved efficiency, enhanced syngas quality, and lower operational costs while further reducing GHG emissions. Recent studies highlight the synergistic effects of co-gasifying biomass and plastics, enhancing energy conversion efficiency and addressing environmental issues often linked to plastic gasification [8]. Despite its potential, research on biomass-plastic co-gasification remains in its early stages, indicating the need for further exploration. Industrial-scale deployment has been limited by the complexity of process parameters that affect performance. Accurately modeling these dynamics using computational fluid dynamics remains challenging, requiring extensive experimentation to optimize, control, and scale up the process [9]. With limited time and resources, researchers often struggle to explore the high-dimensional parameter space experimentally. Therefore, it is essential to optimize the impact of key process parameters on hydrogen production using the available limited experimental data.

With the rapid advancements of the 4th industrial revolution, machine learning (ML), a branch of artificial intelligence, has become a powerful tool for optimizing hydrogen production [10]. ML is particularly effective in uncovering patterns within co-gasification datasets, bypassing the need for a deep understanding of complex physicochemical processes. By employing advanced computational methods, ML can accurately model chemical reactions that are otherwise difficult to capture mathematically [11]. While ML has been widely used to simulate gasification processes [12], identifying the most reliable and interpretable ML algorithm for optimizing hydrogen production remains a key challenge.

Notably, no previous studies have comprehensively evaluated both the performance and interpretability of ML models for predicting hydrogen production from biomass–plastics co-gasification, especially using limited experimental data. Recent comparisons of deep learning (DL) models for hydrogen production prediction have shown surprisingly strong results in small datasets [13], prompting further investigation into how ML models perform in similar conditions. This study seeks to fill this gap by analyzing the performance of various explainable ML models on small co-gasification datasets, aiming to identify the optimal process parameters for maximizing hydrogen production. Such insights are critical for advancing the commercialization of this technology, reducing GHG emissions, and meeting the increasing demand for cleaner fuel alternatives.

To fill this gap in knowledge, this is the first study that delves into investigating a wide range of ML regression models for estimating hydrogen production accuracy from biomass–plastics co-gasification processes. The ML models selected for comparison include linear regression (LR), k-nearest neighbor (KNN), decision tree regression (DTR), random forest regression (RFR), gradient-boosting regression (GBR), SVR, and multilayer perceptron (MLP). The rationale for selecting these ML models was based on their strong performance reported in the relevant literature [14]. Furthermore, they are widely utilized in the field of biomass conversion. These models are assessed using the currently available limited amount of experimental data. All models are configured using a standardized model configuration to maintain consistent model complexity and enable a meaningful comparison. The comprehensive assessment results illustrate the respective benefits and generalization ability of developed regression models in precisely capturing the intricate connection between the process parameters and hydrogen production with regard to the biomass–plastics co-gasification process.

The outcome of this research is expected to hold considerable importance in terms of enhancing and optimizing co-gasification systems involving biomass and plastics. Such advancements could result in increased efficiency and efficacy in the utilization of these invaluable resources. This knowledge has significant promise for promoting the advancement of economically efficient and environmentally friendly co-gasification systems for biomass–plastics. These findings can help researchers and business executives maximize the advantages of biomass–plastics co-gasification while reducing its negative effects on the environment.

## 2. Machine Learning Models

This section provides an overview of the six non-linear ML models selected for evaluation in this study. It highlights the rationale behind their selection, explains their working principles, and discusses their relevance to predicting hydrogen yield in the co-gasification process. The models examined include both parametric and non-parametric techniques, ensuring a comprehensive assessment of their predictive performance.

### 2.1. Linear Regression (LR)

Linear Regression is one of the simplest and most widely used machine learning models for predictive analysis. It establishes a linear relationship between the independent variables (process parameters) and the dependent variable (hydrogen yield) by minimizing the residual sum of squares between observed and predicted values. Mathematically, the model can be represented as follows:

(1) $$Y=\beta_{0}+\beta_{1}X_{1}+\beta_{2}X_{2}+\cdots +\beta_{n}X_{n}+\epsilon$$

where Y denotes the predicted hydrogen yield, $\beta_{0}$ is the intercept, $\beta_{1},\beta_{2},\beta_{3}\ldots \beta_{n}$ are the coefficients representing the contribution of each feature, $X_{1},X_{2},X_{3}\ldots X_{n}$ are the input features (such as temperature, RSS size, HDPE size, and plastics), and ϵ accounts for the error term. Although LR offers a simple and interpretable approach, it is included in the evaluation to assess whether the relationship between process parameters and hydrogen production follows a linear pattern or exhibits non-linear characteristics. This allows for a baseline comparison against more advanced non-linear models, helping to confirm the nature of interactions within the co-gasification process.

### 2.2. k-Nearest Neighbor (KNN) Regression

KNN regression is a non-parametric ML technique that is widely recognized for its simplicity and ease of implementation [15]. This approach is mostly used when there is a lack of prior knowledge regarding the distribution of data. The implementation of KNN utilizes instance-based learning, which operates on the fundamental principle that the nearest neighbors have the greatest potential to impact the prediction. Against this background, the approach uses the output of its neighbors to predict the outcome of the test sample rather than learning the mapping from the training set. Hence, choosing a method to calculate the distance between test and training data is the first step in developing this algorithm. Euclidean distance is commonly observed in this context.

KNN is regarded as a lazy learner, as it saves the training data and starts the learning process when test data are presented for prediction. For instance, to determine which training dataset samples are the k-nearest to a given test sample X, KNN calculates the distance $d_{i}$ between X and each sample $x_{i}$ in the training dataset $D=\left(x_{1},y_{1}\right),\left(x_{2},y_{2}\right),\ldots ,\left(x_{N},y_{N}\right)$. It then picks the k samples with the shortest distance. Finally, it provides the k-nearest neighbor’s weighted average as follows [15],

(2) $$\hat{Y}=\sum_{i=1}^{k}y_{i}\left(X\right)$$

Two key aspects impact prediction results: k value, and distance measuring method. Small k values make outcomes vulnerable to adjacent noise. If k is large, irrelevant points can be considered. The optimal k value is usually computed via cross-validation (CV).

### 2.3. Decision Tree Regression (DTR)

Recently, DTR has grown popular due to its ease of implementation, interpretability, and low computing cost. In contrast to LR models that utilize a single regression function, whether parametric or non-parametric, across the entire dataset and incorporate all independent variables as predictors, the DTR model employs stratified regression analysis and applies different regression models to stratified samples of the independent variables with varying relationships to the dependent variable. More crucially, they can handle non-linear interactions between features, which many other ML algorithms cannot, and find the most essential features that influence decision-making.

The fundamental principle underlying the use of DTR is to divide complicated decisions into simpler ones and create easier-to-interpret predictions. To this end, DTR employs a recursive partitioning of training samples $D=\left(x_{1},y_{1}\right),\left(x_{2},y_{2}\right),\ldots ,\left(x_{N},y_{N}\right)$ into homogeneous subsets at each node based on the partitioning criteria such as Information gain and Gini impurity [16]. This partitioning is carried out by dividing the predicator space into distinct regions $R_{j}$ (where j = 1 to J) that represent the terminal nodes. Each region j is assigned with a constant $\gamma_{j}$ and the tree $\phi_{T_{P}}$ is mathematically modelled as follows [16]:

(3) $$\hat{Y}=\phi_{T_{P}}\left(X,\theta \right)=\sum_{j=1}^{J}\gamma_{j}\;I\left(X\in R_{j}\right)\;$$

where

$$I\left(X\in R_{j}\right)=\left\{\begin{array}{cc} 1, & \mathrm{if}\;X\in R_{j}\\ 0, & \mathrm{otherwise} \end{array}\right.$$

The efficacy of the DTR model relies on the process of identifying the optimal hyperparameter θ through the minimization of the prediction error, which is mathematically represented as follows:

(4) $$\hat{\theta }=argmin_{\theta }\sum_{i=1}^{N}L\left(y_{i},{\hat{y}}_{i}\right)$$

where $y_{i}$ is the outcome of the input sample $x_{i}$ and L(.) is the loss function. For a regression problem, the loss function is mean square error. $\hat{\theta }$ represents the optimized hyperparameter. Against this background, the DTR may overfit if the depth of the tree is extremely high by learning too fine training data details. This phenomenon has the potential to result in generalization on unseen data. Conversely, an extreme low tree depth may lead to underfitting. Therefore, the tuning of tree depth is of utmost importance. Although decision trees have some limitations, they may successfully address the issue of missing data by employing techniques such as weighted impurity and attribute splitting.

### 2.4. Support Vector Regression (SVR)

Support vector machine (SVM) is among the most popular ML algorithms for classification problems on the basis of statistical learning theory. SVR is a modified version of SVM that has been specifically developed to address high dimensionality, nonlinearity, and limited sample sizes in regression problems. In contrast to previous regression models, SVR focuses on minimizing the generalization error instead of minimizing the sum of squared errors between predicted and actual outcomes [16]. In addition, it utilizes the advantages of kernel functions to effectively capture and represent non-linear correlations between the input and output data in a higher-dimensional space. SVR eventually achieves generalized regression efficiency by adequately minimizing both the observed distribution error and training error.

In a broad context, SVR endeavors to identify a function that adequately captures the correlation between $x_{i}$ and $y_{i}$ within the provided training dataset $D=\{\left(x_{1},y_{1}\right),\left(x_{2},y_{2}\right),\ldots ,$$\left(x_{N},y_{N})\right\}$, as seen below [16]:

(5) $$\hat{Y}=\sum_{i=1}^{N}\alpha_{i}\,K\left(x_{i},x_{j}\right)+b\;$$

where

(6) $$K\left(x_{i},x_{j}\right)=\exp\frac{∥x_{i}-x_{j}{∥}^{2}}{2\sigma^{2}}$$

In this case, αi and b are the support vectors and bias term, respectively, determined during training. The kernel function, denoted as K, aims to map the feature space onto a higher dimension. As a result, features that are not linearly separable in lower dimensions can achieve linear separability in higher dimensions. The selection of kernel functions is a crucial task in SVR, and the primary kernel functions used within the SVR framework include, namely linear, polynomial, sigmoid, and radial basis function (RBF). This study uses RBF kernel defined in Equation (6).

Following the selection of a kernel, the empirical risk minimization strategy can be leveraged using a robust insensitive loss function given in Equation (8) to train SVR and achieve an optimal solution. Thus, the support vectors and bias determined by minimizing Equation (7) can subsequently be utilized to make predictions using Equation (5).

(7) $$J=\sum_{i=1}^{m}L_{\epsilon }\left(\hat{y_{i}},y_{i}\right)$$

(8) $$L_{\epsilon }=\left\{\begin{array}{cc} 0, & \mathrm{if}\;y_{i}-\hat{y_{i}}\leq \epsilon \\ y_{i}-\hat{y_{i}}-\epsilon , & \mathrm{otherwise} \end{array}\right.$$

### 2.5. Gradient-Boosting Regression (GBR)

GBR is an ensemble variant of DTR proposed by Friedman. It incorporates a boosting learning approach and the gradient descent method to effectively identify the limits of weak learners and enhance their prediction accuracy. MART, an acronym for Multiple Additive Regression Trees, is a specialized version of gradient boosting that has been tailored exclusively for regression purposes [17]. In general, boosting methods consist of three fundamental components: an ensemble model, weak learners, and a loss function. In this context, weak learners refer to models that exhibit a strong bias with the training dataset and produce outputs that are not noteworthy. Unlike RFR, GBR redefines boosting as a numerical optimization problem and iteratively adds a weak learner to the ensemble model using gradient descent to minimize the loss function. Mathematically, GBR model over M trees can be described as [17],

(9) $$\hat{Y}=G_{m}\left(X\right)=G_{m-1}\left(X\right)+\alpha \phi_{T_{P}\left(X,\theta \right)}$$

Here $T(x,\theta_{m})$ is the weak classifier generated in *m*th iteration, which $\theta_{m}$ is determined by minimizing the loss function, which can be described as

(10) $${\hat{\theta }}_{m}=argmin_{\theta }\sum_{i=1}^{N}L\left(y_{i},{\hat{y}}_{i}\right)$$

where $G_{m-1}\left(X\right)$ is the previous tree residue and GBR minimizes the ${\hat{\theta }}_{m}$ to establish the parameters of the resulting ensemble. The training process seeks to lower the loss function as much as possible to find the local or global optimal solution. Thus, GBR can help reduce bias and variation in prediction results, particularly when applied to small datasets. Therefore, this study utilized GBR to predict hydrogen production in a small-dataset environment.

### 2.6. Random Forest Regression (RFR)

RFR is another ensemble variation of the DTR model that was introduced by Breiman with the aim of improving the performance of decision tree models leveraging the bagging ensemble learning method [18]. Within this learning framework, RFR improves DTR generalization by incorporating randomness at two levels. First, the tree construction process begins utilizing bootstrap sampling with replacement to randomly select the training datasets $T_{b}$ (where b = 1 to B) from the complete training dataset T. Second, during the tree partitioning process, RFR endeavors to identify the most favorable partition by either examining the whole predicator space or by utilizing the maximum number of predicator variables m. Mathematically, the final random forest with B trees $T_{b}$ (where b = 1 to B) is represented as follows [18]:

(11) $$\hat{Y}=\Phi_{T_{B,P}}\left(X\right)=\sum_{b=1}^{B}\phi_{T_{b,m}\left(X\right)}$$

Thus, RFR gains potential to eliminate DTR overfitting by leveraging the benefits of ensemble learning and random sampling. Furthermore, the RFR inherent CV ability with bootstrapped samples estimates realistic prediction error during training, making it appropriate for real-time application. Nevertheless, in real practice, the RF structure must be managed with an adequate amount of trees to balance accuracy and computational burden.

### 2.7. Multilayer Perceptron (MLP)

MLP is a specific category of artificial neural network. It emulates the neural structure of the human brain to address a problem and can be employed for regression or classification tasks. The success of MLP-based neural networks in a wide range of applications depends on their ability to accurately approximate any function to any desired level of accuracy [19]. The MLP model consists of three sorts of layers: an input layer, one or more hidden layers, and an output layer. The nodes within each layer are interconnected by weighted connections to minimize the discrepancy between the output of the network and the desired output. The output signals of an MLP are determined by the summation of the inputs from the previous layer, which is then adjusted by a basic nonlinear activation function as given below [19],

(12) $$\hat{Y}=Act\_fun\left(\sum_{N=1}^{f}W_{N.l}X_{N}\right)$$

In this context, the variables *f* and *l* represent the input process parameters and the number of nodes, respectively. Additionally, a wide variety of activation functions that can be used include linear, sigmoid, softmax, tanh, and rectified linear unit (RELU). In general, the backpropagation strategy is used to optimize the model parameters during the training process. This is carried out by modifying the bias and weights at each epoch, gradually reducing the output error as described in Equation (12). Thus, the strategy facilitates MLP to achieve enhanced precision.

## 3. Model Design and Implementation

**Figure 1:**
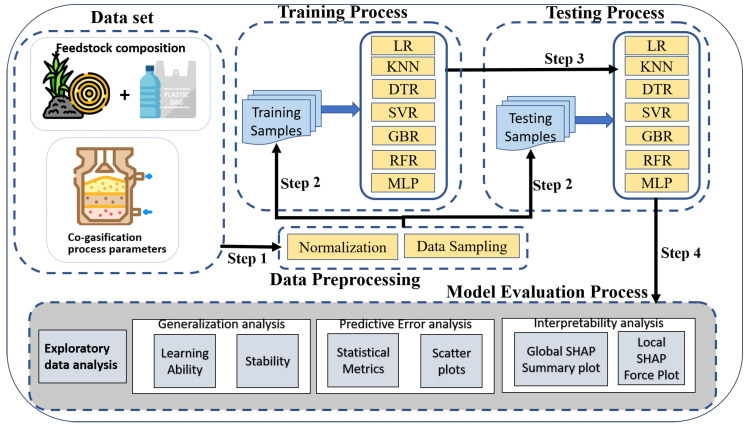
Evaluation framework for explainable ML models in hydrogen yield prediction.

The evaluation process framework is depicted in Figure 1. The figure depicts the relationship between techniques and practices discussed in Section 2. The step-wise exploration of the methodology adopted for comprehensive analysis of the selected predictive models for hydrogen production is as follows:

### 3.1. Data Description

The research data utilized in this study was sourced from the prior literature on the co-gasification of waste plastic and rubber, as documented by [20,21]. The dataset comprises 30 independent experiments carried out in a central composite design, wherein the independent variables include HDPE particles, rubber seed shell (RSS) biomass particle size, plastic quantity in the mixture, and gasification temperature. The quantity of hydrogen generated during the co-gasification of plastic and rubber waste was measured as the dependent variable.

The co-gasification process can be generally expressed by the following chemical equation:

(13) $$C_{x}H_{y}+O_{2}+H_{2}O\to H_{2}+CO+CO_{2}+CH_{4}$$

This equation represents the thermochemical conversion of hydrocarbon-rich feedstocks, such as biomass and plastics, into hydrogen-rich syngas through reactions with oxygen and steam.

The experiment was conducted using a thermogravimetric analyzer that was connected to a mass spectrometer. Table 1 displays the descriptive statistics of the data. The HDPE particle size, biomass RSS particle size, plastic composition in the mixture, and gasification temperature were measured in a range from 0.13 to 0.63 mm, 0% to 40% and 500 °C to 900 °C, respectively.

Table 2 presents the characteristics of the primary raw materials used in this experiment. Here, the elemental analyzer and thermogravimetry analyzer, respectively, were used to perform the ultimate and proximate analyses of these feedstocks. As illustrated in Table 2, the proximate analysis reports the elemental composition of volatile matter (V), fixed carbon (FC), ash (A), and moisture content (M) in the raw materials, while ultimate analysis determines the elemental composition of carbon, hydrogen, nitrogen, and oxygen content in the raw materials. Additional information regarding the dataset can be found elsewhere [21].

### 3.2. Data Preparation

Data preparation is a crucial step that must be undertaken prior to model training. This process guarantees optimal performance and promotes competence in the developed ML regression models. The data format must be consistent for ML algorithms. To achieve this, feature scaling, the process of ensuring that all features are normalized to a consistent range, is essential in data preprocessing to reduce model complexity and accelerate the learning process. It also enables every feature to contribute evenly and prevents model bias. As discussed in Section 3.1, the features collected from the co-gasification process have different scales, different distributions, and sometimes outliers. This study uses min–max normalization to normalize all features, as defined below [22]:

(14) $$\bar{x}=\frac{x-min(x)}{max(x)-min(x)}$$

### 3.3. Model Implementation

The ML models chosen for investigation in this study are developed using the sklearn library in Python. Apart from that, specifically, Python libraries such as statsmodel, seaborn, and matplotlib were utilized for conducting exploratory data analysis with the purpose of gaining insights into the relationship between the predictors and the target variable. Furthermore, this study made use of the Jupyter notebook interface provided by the Google Colaboratory platform [23]. This interface offers a highly interactive programming environment for Python, eliminating the need for local system setup. All tests in this study were conducted using this platform. As stated in the literature, the efficacy of an ML algorithm is dependent upon the quality of the training and testing data employed during the model development process. The diversified training and testing datasets are essential for accurately assessing the true performance of a model, since they mitigate the potential biases that may arise from overfitting or underfitting the model to the training data. Taking into account this fact, stratified sampling was adopted to split the study dataset using the most common split ratio of 80:20. This indicates that 80% of the data are used for training and the remaining 20% for the testing set [24].

Following the process of data splitting, the initial step involves the implementation of selected ML models with baseline hyperparameter configurations. These models are then trained using a 10-fold cross validation (10-CV) technique, which aims to provide more reliable and stable estimates. The aforementioned models are labeled as unoptimized ML models. For the second task, we reimplemented all models, specifying the hyperparameter space for each ML model. Subsequently, a 10-CV was utilized to determine the optimal hyperparameters for all models. The ML models developed using the optimal hyperparameters have been designated as tuned models.

### 3.4. Model Hyperparameter Tuning

Hyperparameters refer to the parameters that are used to define the architecture of a model. The process of selecting appropriate hyperparameter values for a given ML algorithm and dataset is crucial [25]. Hyperparameters play a significant role in controlling the model learning process and can greatly affect the effectiveness of ML models. The objective of hyperparameter tuning is to enhance the generalization performance of the model by identifying the optimal values for the hyperparameters that yield the most favorable out-of-sample performance.

The cross-validated grid search function (GridSearchCV) in python is employed with negated MAE as a scoring parameter to analyze every possible combination of hyperparameters and determine the optimal set that maximizes generalization performance. Finally, to evaluate how well the best-found combination generalizes, we measured its score on the hold-out test set. Table 3 provides insight into the hyperparameter space for all ML models.

## 4. Results and Discussion

The assessment of the developed ML models is of utmost importance in order to select the ideal model for accurate prediction of hydrogen production in a co-gasification process. Within this framework, experiments are designed to evaluate the developed models across three dimensions, such as its generalization ability, prediction error, and interpretability. This section presents the results analysis to compare the effectiveness of the seven regression models for the prediction of hydrogen production. Finally, this section concludes by presenting the most important contribution made by the models chosen in this study.

### 4.1. Exploratory Data Analysis (EDA)

**Figure 2:**
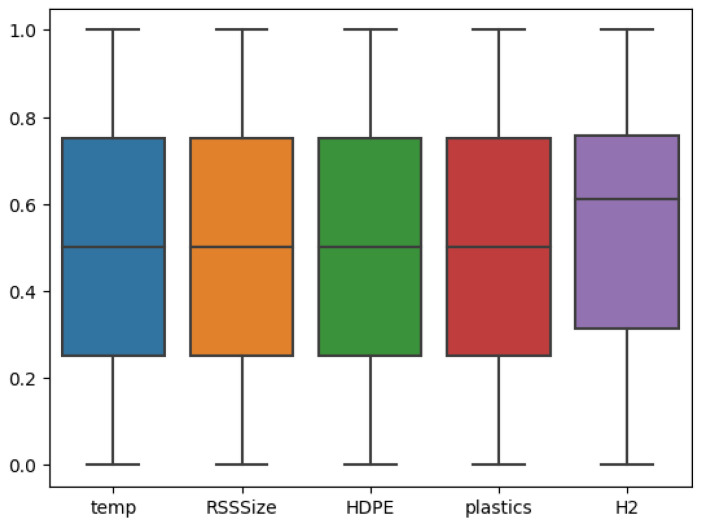
Box plot for outlier analysis.

As a first step, the research data presented in the data description section is preprocessed based on the procedure outlined in Section 3. Subsequently, the preprocessed data was subjected to analysis using a statistical technique known as EDA. Briefly, EDA is a very important step in ML that needs to be conducted before model development to understand and prepare the study data [26]. In EDA, the first task is to examine the presence of outliers in the modeling dataset and to investigate the data homogeneity. For this purpose, Python’s boxplot is shown in Figure 2. Observing these results, it is obvious that the dataset has no outliers and that the distribution of all input features of interest is in a reasonable range, making them suitable for developing the predictive models.

**Figure 3:**
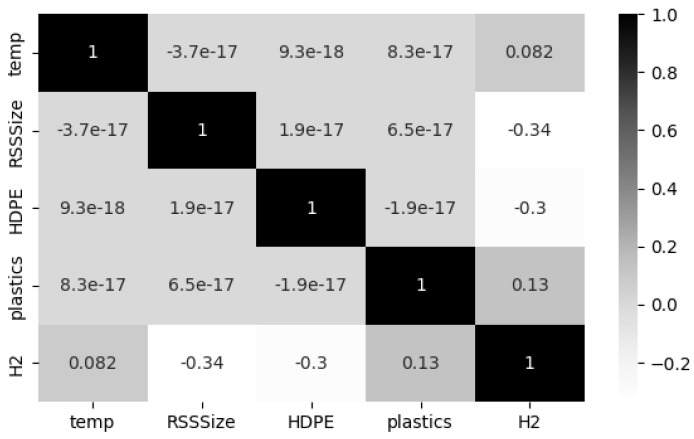
Heat map for correlation analysis.

Furthermore, a Pearson Correlation Coefficient (PCC) analysis was conducted to unveil the relationships among all parameters in the co-gasification process. This process enables us to identify the co-linearity between input features and eradicate the overlapping effect of input features. Ideally, |PCC|=1 represents strong data correlation, whereas |PCC|=0 signifies the absence of correlation. Furthermore, it is a standard practice to consider only one variable for model development when the |PCC| between two variables is greater than 0.6 [27]. The Seaborn heatmap illustrated in Figure 3. demonstrates the degree of dependency between the input process parameters and the target variable hydrogen production. From careful observation of the figure, it is evident that the correlation coefficients between the four process parameters is <0.01, declaring they are not correlated to each other. Also, it is worthy to observe that the operating temperature and quantity of plastics show a positive correlation with hydrogen production, with a correction coefficient of 0.082 and 0.13, respectively. Whereas the other two process parameters, such as RSS size and HDPE size, show a negative correlation on hydrogen production with a coefficient of −0.34 and −0.3, respectively. These observations imply that all these four process parameters have varying influences on hydrogen production and are required to be considered for model development.

### 4.2. Model Generalization Analysis

After ensuring the quality of the study data and acquiring a thorough understanding of its characteristics from EDA analysis, the chosen seven different ML models are developed and trained following the procedures outlined in Section 3. The primary objective of the first set of experimental analyses is to investigate the potential of the developed ML models for their generalization ability on the available research data. This investigation is conducted from two distinct perspectives, which will be delineated in the following sections.

#### 4.2.1. Model Learning Ability

**Figure 4:**
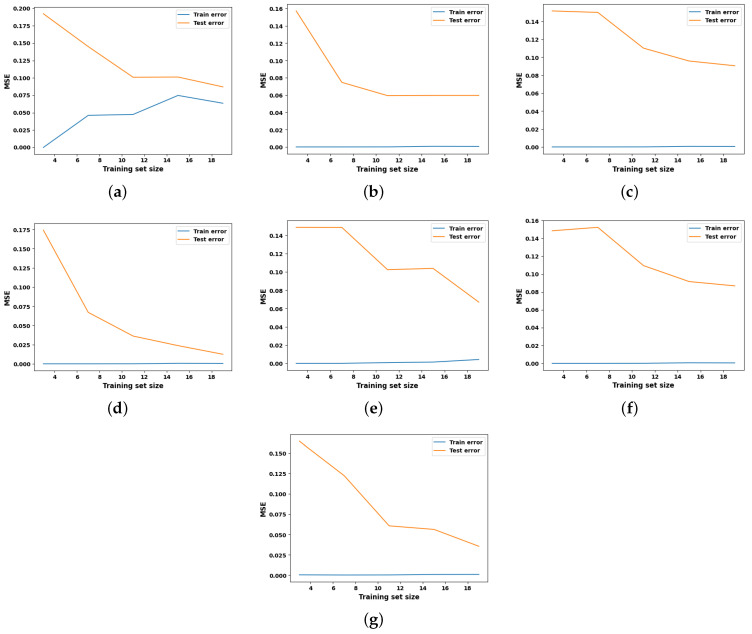
Learning curve analysis of all explainable ML models with optimized hyperparameters. (**a**) LR, (**b**) KNN, (**c**) DTR, (**d**) SVR, (**e**) GBR, (**f**) RFR, (**g**) MLP.

**Figure 5:**
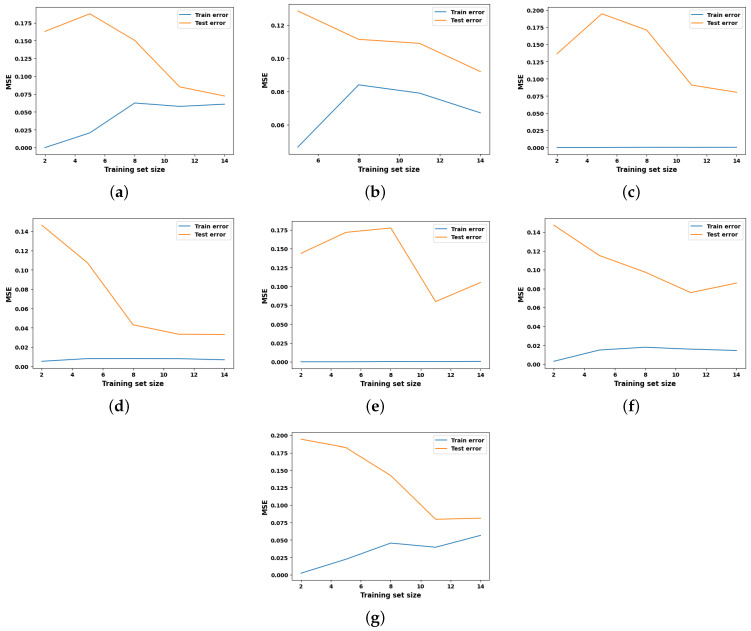
Learning curve analysis of all explainable ML models with default hyperparameters. (**a**) LR, (**b**) KNN, (**c**) DTR, (**d**) SVR, (**e**) GBR, (**f**) RFR, (**g**) MLP.

Learning curves are widely recognized as a valuable tool in ML for visually representing the training progress over time. The scholarly literature suggests utilizing learning curves to analyze ML models, as they can assist in identifying overfitting or underfitting and can contribute to improving the performance of the models [28]. Acknowledging the benefits of the learning curves, it is employed in this study to investigate the performance of the ML models for hydrogen prediction in the co-gasification process. The corresponding results for the seven ML models with hyperparameter optimization are depicted in Figure 4, while their respective baselines are shown in Figure 5. The learning curve is shown by two solid lines: blue for the training data, and orange for the testing data. In this context, the MSE is utilized to evaluate the predictive performance of the models as they advance in their learning process. In general, the errors observed on the testing dataset serve as indicators of model generalization, whereas errors observed on the training dataset provide insights into the goodness-of-fit. Consequently, researchers recommend generalization as a ‘gold standard’ for model selection, as it reflects the model’s ability to adapt to new data and make accurate predictions.

Keeping this in mind, and observing the MSE values in Figure 4 and Figure 5, it is evident that the learning curves of all the competing models, except for KNN and MLP, exhibit only minor differences in hyperparameter optimization. This finding confirms that the hyperparameter optimization does not have a substantial influence on predictive performance for the dataset under examination. A careful examination of the learning curves for all optimized models reveals distinct trends in MSE minimization as the number of training samples increases. For example, SVR and MLP demonstrate the strongest generalization capabilities, achieving the lowest test MSE values of 0.025 after optimization, showcasing their effectiveness in capturing complex non-linear patterns. GBR also exhibits good generalization, with significant improvements in test error after optimization; however, its performance remains slightly below that of SVR and MLP due to limitations in capturing more complex relationships. KNN, RFR, and DTR display moderate performance, with stable training errors and varying improvements in generalization as the dataset size increases. LR, on the other hand, consistently exhibits high training and test errors, reflecting its inability to model non-linear relationships and its unsuitability for this problem. Overall, SVR and MLP perform the best, achieving the highest generalization and predictive accuracy. GBR demonstrates good but slightly moderate performance compared to the top models, benefiting from regularization and optimization. KNN, RFR, and DTR show moderate performance improvements with larger datasets. LR performs the weakest, failing to adapt to the non-linear complexities of the data.

#### 4.2.2. Model Stability

Model stability that assesses how consistently a ML model makes accurate predictions across training data is of utmost importance to draw an informed decision regarding model selection. Currently, CV is widely accepted in the field of ML and data analysis and is considered a universal tool to assess the stability of a model to generalize beyond its training set [29]. More importantly, the variance of CV serves as a stable error measurement to assess how accurately the model prediction generalizes over a set of independent data. If the variance is low, then the model can be deemed stable and considered the most suitable model for accurate prediction.

The primary attraction for CV encompasses three distinct aspects: First, its simplicity to use. Second, its sensitivity to the functional form dimension of model complexity, in contrast to other generalization criteria such as Akaike and Bayesian Information Criterion. Third, it offers a confidence measure for estimating generalization error, especially when the training set used to develop a ML model is small. Therefore, it is indeed imperative to utilize CV within a co-gasification framework, considering the challenges and costs of acquiring large datasets in this framework.

**Figure 6:**
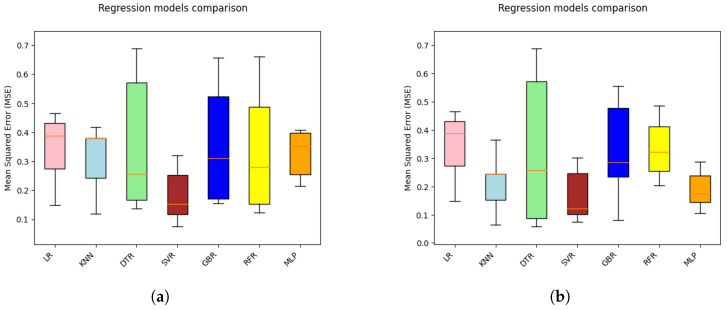
Box Plot of 5-CV results for all explainable ML models in hydrogen yield prediction. (**a**) Default hyperparameters, (**b**) Optimized hyperparameters.

Realizing its benefits, this research work employs CV with MSE as its objective function to compare the stability of the developed ML models. Next, the mean and standard deviation (std) over the 5-fold CV (5-CV) results of all the ML models developed with default and optimal hyperparameters are presented in Table 4. In order to enhance the comprehensibility of the findings, a visual representation of the CV results is illustrated using the box plot in Figure 6.

Observing the mean and std of CV results, it is evident that only the SVR model displays the lowest CV score with a mean and std of 0.18 and 0.09, respectively. Conversely, all other ML models yield relatively larger CV scores. While comparing the CV results of all ML models trained with default and optimized hyperparameters, it is apparent that, except for LR and DTR, all other ML models demonstrate enhanced prediction performance with hyperparameter optimization.

Visual inspection of the box plot clearly indicates that SVR presents a comparatively lower median value than other competing ML models. Also, it can be seen that KNN and MLP models with tuned hyperparameters display smaller boxes compared to other models and conform to their stable performance, with minor variations in results across the folds. This finding is consistent with the learning curve behavior illustrated in Figure 4.

### 4.3. Prediction Performance Analysis

The second set of experimental analyses aims to evaluate and compare the efficacy of the developed ML models for predicting hydrogen production in the co-gasification process. In this direction, experiments were devised to train the developed ML models using the training set that comprises 80% of the research dataset. The trained ML models were subsequently assessed using the testing set created with the remaining 20% of the research data. The prediction performance measured on the testing set are presented and compared both quantitatively and qualitatively, as follows.

#### 4.3.1. Quantitative Statistical Metrics

**Figure 7:**
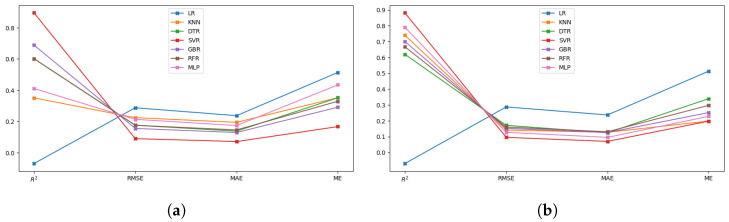
Line graph illustrating the statistical performance metrics for all explainable ML models. (**a**) Default hyperparameters, (**b**) Optimized hyperparameters.

The best-performing model cannot be determined by merely comparing the models using a single assessment metric. Consequently, at this phase of analysis, the prediction performances of the developed ML models are compared, employing four different statistical measures, namely, coefficient of determination $\left(R^{2}\right)$, root mean squared error (RMSE), mean absolute error (MAE), and max error (ME), to examine the correlation between the predicted test results and the measured hydrogen yield [30]. Table 5 presents these statistical measures as a standard indicator to compare and analyze the performance of all the analyzing ML models with default and optimized hyperparameters for hydrogen yield prediction in co-gasification. Figure 7 displays a line graph of the same data for easier interpretation.

In this context, $R^{2}$ values closer to unity, along with reduced values of RMSE, MAE, and ME, are recognized as key performance indicators for an idle model with improved prediction performance. In this viewpoint, comparing the results between default and optimized hyperparameters reveals that the optimization process enhances the key performance indicators across all prediction models, except for LR. This finding supports two claims: First, the importance of hyperparameter optimization while developing a prediction model for the co-gasification process. Second, the accuracy of the hyperparameter optimization procedure is validated by the good fit exhibited by all non-linear ML models for the co-gasification process. Furthermore, the limited performance of the LR model, indicated by a negative $R^{2}$, reflects its inability to capture the complex non-linear relationships present in the dataset. A negative $R^{2}$ suggests that the model’s predictions are less accurate than a simple baseline, such as predicting the mean of the target values. This underscores the inadequacy of linear models for the co-gasification process and further reinforces the need for non-linear approaches to accurately model the intricate dependencies between input parameters and hydrogen yield.

From the vertical comparison of the results through different prediction models with optimized hyperparameters, the lowered statistical error measures validate that the predictive models have successfully captured the impact of all input parameters to accurately predict the hydrogen yield in the co-gasification process. In particular, SVR appears to be the most potential prediction model with the lowest error values (RMSE, MAE, and ME) and highest $R^{2}$ value of 0.9. MLP follows the same trend and achieves the second-best prediction performance with a $R^{2}$ greater than 0.8 compared to other competing ML models. Although GBR and KNN failed to exhibit good generalization ability when cross-validated with the whole research data, their prediction performance on the 20% testing set is deemed satisfactory with $R^{2}>0.7$. This finding suggests that, by strengthening the training dataset, the predictive performance of these non-linear models can be enhanced.

#### 4.3.2. Qualitative Scatter Plot

Scatter plots, together with a regression line, are widely acknowledged as a versatile and very valuable method for visually and statistically evaluating the correlation between model predictions and the observed values [31]. They are commonly employed as a primary approach to examine the accuracy of model predictions within data-driven research. In light of this foundation, the present study employs a scatter plot-based regression analysis to provide more empirical evidence for the findings obtained in the previous sections.

**Figure 8:**
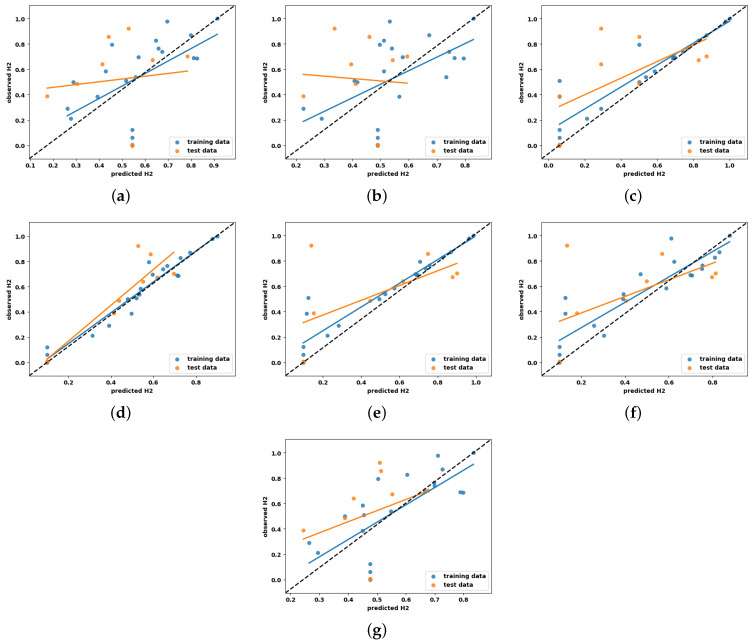
Scatter plots of all explainable ML models with default hyperparameters for hydrogen yield prediction analysis. (**a**) LR, (**b**) KNN, (**c**) DTR, (**d**) SVR, (**e**) GBR, (**f**) RFR, (**g**) MLP.

**Figure 9:**
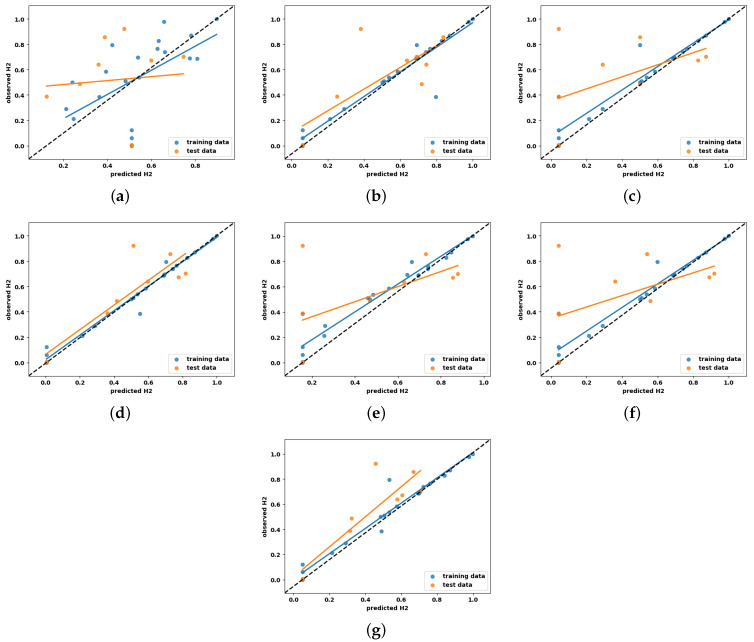
Scatter plots of all explainable ML models with optimized hyperparameters for hydrogen yield prediction analysis. (**a**) LR, (**b**) KNN, (**c**) DTR, (**d**) SVR, (**e**) GBR, (**f**) RFR, (**g**) MLP.

Here, the vertical distance from the regression line to a specific point indicates the prediction error for that sample. As a result, a prediction model deemed effective exhibits minimal errors, and their predictions tend to cluster around the regression line. For an ideal regression model, their predictions align perfectly with the actual measurements, resulting in all data points falling precisely along the diagonal line known as the [1:1] regression line. The scatter plots illustrating the experimentally observed and predicted hydrogen yield for the ML models developed with default and tuned hyperparameters are shown in Figure 8 and Figure 9, respectively. Here, the [1:1] regression line is shown by the black dashed line at a 45° angle, and the data distribution represented by the blue and orange colors corresponds to the training and testing predictions, respectively.

Analyzing the figures in Figure 8 and Figure 9, it is evident that LR exhibits more scattered prediction with the testing and training set. The observed inferior performance of LR in comparison to other ML models further substantiates the non-linear behavior of hydrogen production with the process parameters. One unexpected observation is that DTR and RFR, which showed overfit on the training set in the learning curve, have shown scattered predictions even with the training set. This condition may be attributed to the hard rule-based learning approach employed by the DTR and RFR. Another notable observation is that, with the exception of DTR and RFR, all other ML models exhibit a close clustering of predictions around the regression line. This finding adds more support for their ability to effectively describe the non-linear correlation between hydrogen production and the process parameters.

### 4.4. Model Interpretation

In practice, if a researcher finds a ML model with an acceptable level of accuracy, the subsequent step involves gaining insight into the prediction process and making informed decisions in light of expert domain knowledge. Unfortunately, the black-box mechanism of ML models presents challenges in understanding the impact and influence of input parameters on target within the modeling process. This experimental analysis takes a step further to address the problem at hand by analyzing the interpretability of the developed ML models and rationalizing their predictions, irrespective of their complexity [32]. The post hoc interpretability analysis integrates the Shapley approach with the developed ML models to provide substantial insights into the final prediction of the model from both local and global perspectives [33]. The sections that follow will explore these findings in further detail.

#### 4.4.1. Global Explanation with Summary Plot

Global explanations, or dataset-level interpretations, are essential for understanding the overall relationships between features and model predictions across the entire dataset [34]. SHAP offers an unified framework that facilitates both local and global analysis, making it a valuable tool for interpretability. Unlike surrogate models that approximate the behavior of complex models, SHAP aggregates Shapley values from multiple predictions, providing accurate and consistent feature importance scores [35]. This consistency ensures that global insights derived from SHAP align with the model’s actual predictions, enhancing transparency and trust in the model’s decision-making process. Therefore, the present study employs SHAP to achieve global interpretability across all ML models developed for hydrogen production analysis [36]. The summary plot not only illustrates the significance of features, but also reveals their positive or negative associations with the target outcome.

**Figure 10:**
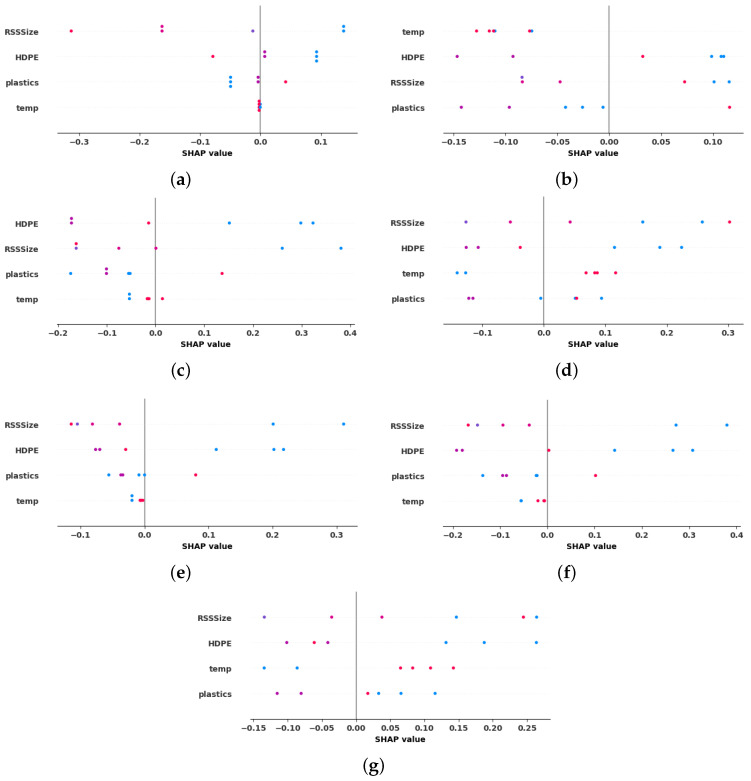
SHAP summary plots of all explainable ML models in hydrogen yield prediction. (**a**) LR, (**b**) KNN, (**c**) DTR, (**d**) SVR, (**e**) GBR, (**f**) RFR, (**g**) MLP.

The SHAP summary plots in Figure 10 provide a clear depiction of how key process parameters influence hydrogen yield during the co-gasification process across various ML models. The x-axis represents the magnitude and direction of feature contributions, where positive values indicate increase in hydrogen production, while negative values reflect a reduction in yield. The y-axis ranks features by importance, with the most influential parameters placed at the top. A color gradient further enhances interpretability, with red dots signifying higher feature values and blue dots representing lower values.

Analysis of the summary plot in Figure 10 reveals that, on average, all process parameters significantly impact hydrogen production during biomass–plastics co-gasification. The widespread distribution of dots across the SHAP plots for these parameters reinforces this observation. Notably, KNN, SVR, and MLP consistently highlight HDPE size, RSS size, and temperature as the primary factors driving hydrogen yield, whereas other models place greater emphasis on plastics. This agreement with the existing literature and prior studies reinforces the reliability and accuracy of the results.

A closer examination of the color gradient reveals how variations in temperature, RSS size, and HDPE size influence hydrogen output. Prior studies highlight a non-linear relationship between RSS size and hydrogen yield: moderate RSS values enhance production, while extremely large or small values reduce it. This non-linear trend is effectively captured by SVR, KNN, and MLP, demonstrating their ability to model complex interactions between process parameters in alignment with previous research. However, while KNN successfully models the relationship between RSS size and hydrogen yield, it shows inconsistencies in capturing the effect of temperature on hydrogen production. This is evident from red dots frequently appearing on the negative x-axis, suggesting that KNN may overfit certain features or misinterpret their contributions, leading to deviations from empirical data.

In contrast, SVR and MLP effectively capture the expected relationship between temperature and hydrogen yield, showcasing greater robustness and reliability. Thus these two models provide comprehensive and interpretable explanations that closely align with previously published studies and experimental data on the co-gasification process, reinforcing their ability to model complex interactions between process parameters accurately.

The insights derived from SHAP summary plots provide actionable pathways for optimizing hydrogen production. By fine-tuning critical parameters such as temperature, HDPE size, and RSS size based on SHAP analysis, operators can enhance process efficiency and maximize yield. This data-driven approach effectively bridges the gap between model predictions and real-world implementation, thereby supporting advancements in biomass–plastic co-gasification technologies. In summary, SHAP summary plots offer a comprehensive understanding of the internal mechanics of ML models applied to hydrogen production. By guiding adjustments to process parameters, these plots drive significant improvements in operational performance and sustainability.

#### 4.4.2. Local Explanation with Force Plot

Local explanations offer insights into how individual feature values influence specific model predictions, enabling a more granular understanding of model behavior. SHAP force plots illustrate the forces driving predictions higher or lower relative to a baseline value. This section examines two cases of hydrogen yield—one with low yield, and one with high yield—highlighting how force plot analysis uncovers the impact of critical features on model outputs.

**Figure 11:**
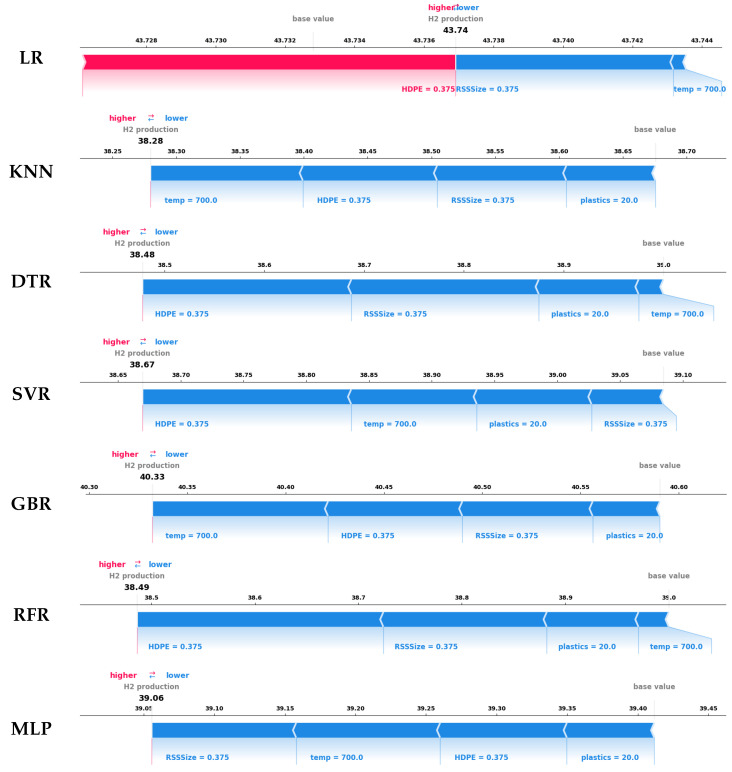
SHAP force plots of all explainable ML models for an instance yielding low hydrogen.

For the low-yield case depicted in Figure 11, the experimentally recorded hydrogen production during the co-gasification experiment is 38.625. SHAP force plots reveal that, with the exception of the LP model, all other ML models correctly predict values below their baselines. This underestimation is primarily driven by lower values of temperature, plastic percentage, and HDPE size—key process parameters that collectively suppress hydrogen yield. Among the models, SVR provides the most accurate prediction of 38.67, closely aligning with the experimental result. The SVR model’s ability to reflect subtle parameter interactions highlights its reliability and precision in modeling low-yield scenarios.

**Figure 12:**
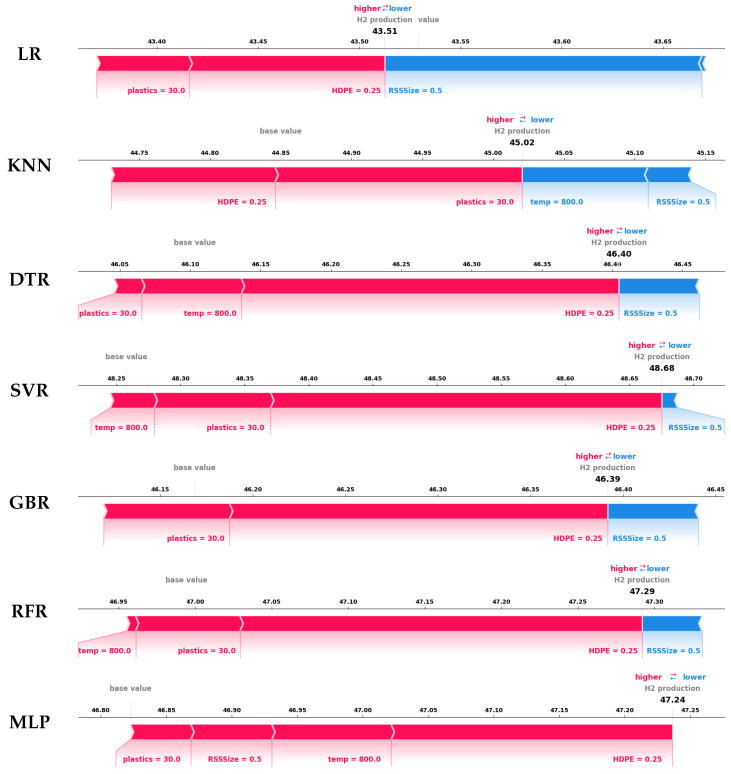
SHAP force plots of all explainable ML models for an instance yielding high hydrogen.

In the high-yield case shown in Figure 12, the experimentally recorded hydrogen production is 48.21; SHAP force plots demonstrate varying predictive performance across different models. A key observation is that LR consistently underperforms, producing predictions below the baseline. This highlights LR’s inability to capture the non-linear relationships between process parameters and hydrogen yield, exposing its limitations in modeling the complex interactions essential for accurate co-gasification process predictions.

Among the non-linear models, KNN and GBR exhibit weaker predictive performance. KNN, with a predicted yield of 45.02, underperforms due to a misinterpretation of temperature, incorrectly associating higher temperatures with reduced hydrogen production. This aligns with KNN’s SHAP summary plot, reinforcing the model’s tendency to misattribute negative contributions to temperature. Similarly, GBR predicts a yield of 46.39, underperforming due to its failure to account for temperature’s role. By neglecting temperature as a key driver, GBR limits its predictive accuracy and ability to match the observed high-yield value.

In contrast, DTR, RFR, MLP, and SVR demonstrate stronger predictive capabilities. For example, the tree-based models, DTR and RFR, yield 46.40 and 47.29, respectively. These models successfully capture the combined influence of temperature, plastics, RSS size, and HDPE size, achieving higher predictive accuracy than KNN and GBR. Their ability to model intricate feature interactions enhances their performance. However, the slight discrepancies between their predictions and the recorded high yield suggest variations in feature prioritization, indicating the potential for overfitting to certain parameters. This overfitting may reduce the stability of their predictions, especially when applied to new datasets or varying conditions.

Likewise, MLP also shows strong performance, predicting a yield of 47.24. While MLP effectively models non-linear dependencies, it underrepresents the negative impact of RSS size. This may result from the model’s sensitivity to feature scaling or weighting, leading to imbalances in feature prioritization. Despite outperforming KNN and GBR, MLP’s inconsistent handling of RSS size lowers its reliability in high-yield scenarios.

Building upon the analysis of non-linear models, SVR emerges as the most accurate model, predicting a yield of 48.68, which closely aligns with or surpasses the experimentally recorded high yield. The force plot for SVR highlights the strong positive contributions of temperature and plastics while accurately accounting for the negative impact of RSS size. Overall, SVR consistently outperforms other models, providing the most accurate and comprehensive predictions. Its ability to model non-linear interactions and account for the influence of all critical process parameters reinforces SVR’s position as the most reliable model for predicting hydrogen yield in the co-gasification process.

### 4.5. Comparative Analysis

This section provides a detailed comparison of the ML models developed in this study, offering insights into their predictive performance, generalization capabilities, and industrial applicability. The analysis begins with a comparison to prior research, addressing discrepancies in performance and overfitting concerns. This is followed by an examination of the strengths, limitations, and suitability of each model for hydrogen yield prediction in the co-gasification process.

#### 4.5.1. Comparison with Related Studies

The performance of the ML models developed in this study for predicting hydrogen yield from the co-gasification of biomass and plastics reveals notable differences compared to previous work. Ayodele et al. [21] and Sheila Devasahayam [13] reported $R^{2}$ exceeding 0.98 using MLP and deep learning networks, respectively. In contrast, the highest $R^{2}$ value attained in this study is 0.86. While this reflects strong predictive accuracy, it falls short of the performance presented in earlier research. A key factor contributing to this discrepancy lies in the evaluation methodologies employed. Prior studies utilized the hold-out method, dividing the dataset into distinct training and testing subsets. While this approach provides a straightforward assessment, it can lead to inflated $R^{2}$ values by focusing predominantly on the training data, potentially masking issues of overfitting and limiting insight into model generalization. In contrast, the present study employs 5-CV, an iterative and robust evaluation method that thoroughly assesses the model’s generalization ability. By partitioning the data into five subsets and validating performance across each fold, this approach minimizes overfitting risks and yields a more reliable estimate of predictive accuracy.

The scatter plots in this study reinforce this by illustrating the correlation between predicted and actual values for both training and testing data points (Figure 8 and Figure 9), highlighting the model’s ability to generalize effectively. Conversely, scatter plots in prior studies typically reflect results based solely on training data, leading to inflated $R^{2}$ values and failing to represent the model’s performance on unseen data accurately. Several additional factors that contribute to the observed differences in results are as follows:

(a) **Model complexity and overfitting mitigation:** The exceptionally high $R^{2}$ values reported in earlier studies may indicate overfitting, particularly given the small dataset size (n = 30). In this study, regularization techniques—such as L1/L2 penalties and early stopping—were applied to mitigate overfitting. Although these measures led to slightly lower $R^{2}$ values, the resulting models demonstrated improved generalization and stability, essential for deployment in real-world scenarios.
(b) **Dataset size and model selection:** The limited dataset size presents challenges for complex models like MLP, which typically require larger datasets to generalize effectively. While MLP excels in capturing non-linear relationships, it is prone to overfitting when trained on small datasets. To address this, simpler models such as SVR were prioritized in this study. SVR, which achieved the highest $R^{2}$ value of 0.86, benefits from kernel functions and built-in regularization, allowing for it to perform reliably with small datasets and enhancing its suitability for hydrogen yield prediction.

In summary, although the models developed in this study exhibit slightly lower $R^{2}$ values compared to prior research, the inclusion of testing data in model evaluation and the implementation of overfitting mitigation techniques ensure greater generalizability. This approach prioritizes model robustness and stability, making the findings more applicable to practical industrial environments.

#### 4.5.2. Comparison of Model Strengths, Limitations, and Industrial Suitability

The comparative analysis of explainable ML models, as summarized in Table 6, highlights the unique strengths, limitations, and industrial applicability of each model. This evaluation provides a roadmap for selecting the most appropriate models for hydrogen yield prediction, balancing predictive performance, interpretability, and scalability.

Among the models assessed, SVR demonstrated the highest level of reliability, consistently achieving strong generalization performance, high predictive accuracy, and excellent interpretability. SVR’s ability to model non-linear relationships, enhanced by regularization techniques such as tuning the penalty parameter (C) and kernel coefficient (gamma), positions it as a leading choice for industrial applications. Its robustness across varying datasets reduces the risk of overfitting, ensuring consistent performance in operational environments. KNN also displayed strong predictive performance by effectively capturing local data patterns and non-linear dependencies. However, KNN’s computational intensity scales with dataset size, raising concerns about scalability for large datasets. CV and optimized k were applied to improve performance, resulting in excellent interpretability and high industrial applicability. The MLP showed significant potential in modeling complex non-linear relationships, but required extensive hyperparameter tuning to minimize overfitting. Techniques such as dropout and early stopping enhanced MLP’s resilience, making it a feasible option for industrial deployment where computational resources are sufficient. Tree-based models, including the DTR and RFR, provided moderate performance, but were prone to overfitting without appropriate regularization. Methods such as pruning, limiting tree depth, and bootstrap aggregation were employed to enhance model generalization. Despite these efforts, the models demonstrated performance variability, indicating that they may be more effective when integrated into hybrid approaches to improve stability and reliability. GBR offered competitive accuracy, but required careful hyperparameter tuning to prevent overfitting. While GBR’s boosting mechanism improved generalization, the iterative training process may pose challenges for real-time industrial deployment. In conclusion, the selection of an ML model for hydrogen yield prediction requires balancing predictive performance, interpretability, and scalability. SVR emerged as the most reliable model, while MLP offer alternative solutions for non-linear modeling, provided overfitting mitigation techniques are applied. These findings underscore the importance of hyperparameter tuning and regularization to ensure robust and scalable model performance, contributing to advancements in co-gasification process optimization.

## 5. Conclusions

This study systematically investigated the performance of seven different ML models, including LR, KNN, DTR, SVR, GBR, RFR, and MLP, for predicting and optimizing hydrogen production based on the process parameters for the biomass-plastics co-gasification process. To this end, exploratory data analysis was conducted as a preliminary step to assess the quality of the study dataset before utilizing it to train the selected ML models. Eventually, after optimizing the hyperparameters of all the trained ML models, a comprehensive set of experiments was devised to evaluate the performance of these models across three dimensions, including generalization ability, prediction capabilities, and interpretability. Based on the results, the following can be concluded:

- The generalization ability analysis conducted through qualitative and quantitative methods using learning curves and CV showed that SVR and MLP models demonstrated superior generalization potential over other competing models, achieving a minimum MSE of approximately 0.025.
- The prediction performance analysis highlights the potential of SVR, with $R^{2}$ value of approximately 0.9, reflecting its strong ability to model the nonlinear relationship between hydrogen production and process parameters.
- The interpretability analysis of the developed ML models at global and local levels using the SHAP summary plot and force plot, respectively, revealed that the SVR and tree-based models were more successful in reliably elucidating the influence of the process parameters on hydrogen production and concurred with both the experimental observations and the previously published literature.

These findings emphasize the importance of selecting appropriate ML models to optimize hydrogen production in the co-gasification process. This study highlights the significance of balancing predictive accuracy with model interpretability and stability, ensuring the successful deployment of ML models in industrial environments. Future work will focus on expanding the dataset and refining ensemble approaches to further enhance model performance and address the challenges associated with small datasets. By integrating explainability into model evaluation, this research provides a robust framework for optimizing the co-gasification process, contributing to the advancement of cleaner and more sustainable hydrogen production technologies.

## Figures and Tables

**Table 1:** Descriptive data statistics.

	Temp °C	RSS Size mm	HDPE mm	Plastics wt. %	H_2_ vol. %
**count**	30.00	30.00	30.00	30.00	30.00
**mean**	700.00	0.38	0.38	20.00	44.71
**std**	90.97	0.11	0.11	9.10	3.65
**min**	500.00	0.12	0.12	0.00	38.57
**25%**	600.00	0.25	0.25	10.00	42.21
**50%**	700.00	0.38	0.38	20.00	45.64
**75%**	800.00	0.50	0.50	30.00	47.33
**max**	900.00	0.62	0.62	40.00	50.12

**Table 2:** Characteristics of HDPE and RSS.

Sample	Proximate Analysis Wt. %, Dry Basis				Ultimate Analysis Wt. %, Dry Basis				
	V	FC	A	M	C	H	N	O	S
**HDPE**	99.46	0	0.34	0	81.45	12.06	0.34	0.79	5.36
**RSS**	80.98	6.62	3.81	8.59	44.31	4.38	0.51	50.67	0.13

**Table 3:** Optimized hyperparameters for all explainable ML models in hydrogen yield prediction.

ML Models	Hyperparameters	Search Range	Selected Value
**KNN**	n_neighbors	[2, 3, 4, 5, 7, 9, 11]	2
	weights	[‘uniform’, ‘distance’]	‘distance’
	metric	[‘minkowski’, ‘euclidean’,‘manhattan’]	‘euclidean’
**DTR**	max_depth	[3, 5, 7, 9, 11, 15]	9
	min_samples_split	[2, 3, 4, 5]	2
	criterion	[‘gini’,‘entropy’]	‘gini’
**SVR**	gamma	[1, 1.5, 2, 2.5]	1.5
	C	[3, 4, 4.5, 5, 5.5]	4.5
	epsilon	[0.1, 0.01, 0.5, 1]	0.01
**GBR**	loss	[‘squared_error’, ‘absolute_error’]	‘squared_error’
	learning_rate	[0.1, 0.01, 0.5, 1]	0.1
	max_depth	[3, 5, 7, 9, 11, 15]	5
	n_estimators	[50, 100, 200, 250, 300]	250
**RFR**	max_depth	[3, 5, 7, 9, 11, 15]	5
	max_features	[2, 3, 4, 5]	4
	criterion	[‘gini’,‘entropy’]	‘gini’
	n_estimators	[10, 15, 20, 25, 30]	15
**MLP**	hidden_layer_sizes	[(100, 50, 25), (50, 25), (50)]	(50)
	learning_rate_init	[0.1, 0.01, 0.5, 1]	0.01
	power_t	[0.1, 0.5, 1]	0.5
	max_iter	[250, 500, 1000]	500

**Table 4:** 5-CV error analysis for all explainable ML models in hydrogen yield prediction.

ML Models	MSE with Default Hyperparameters		MSE with Tuned Hyperparameters	
	Mean	Std	Mean	Std
**LR**	0.34	0.12	0.34	0.12
**KNN**	0.31	0.11	0.23	0.10
**DTR**	0.36	0.22	0.35	0.24
**SVR**	0.18	0.09	0.17	0.09
**GBR**	0.36	0.20	0.32	0.15
**RFR**	0.34	0.20	0.33	0.10
**MLP**	0.33	0.08	0.24	0.09

**Table 5:** Statistical performance metrics for all explainable ML models in hydrogen yield prediction.

ML Models	Hydrogen Yield Prediction with Default Hyperparameters				Hydrogen Yield Prediction with Optimal Hyperparameters			
	*R* ^2^	RMSE	MAE	ME	*R* ^2^	RMSE	MAE	ME
**LR**	−0.07	0.29	0.24	0.51	−0.07	0.29	0.24	0.51
**KNN**	0.35	0.22	0.19	0.35	0.75	0.13	0.12	0.19
**DTR**	0.6	0.17	0.14	0.35	0.64	0.15	0.13	0.28
**SVR**	0.86	0.10	0.09	0.17	0.9	0.09	0.07	0.15
**GBR**	0.56	0.18	0.14	0.35	0.71	0.14	0.13	0.21
**RFR**	0.66	0.16	0.13	0.33	0.68	0.16	0.13	0.3
**MLP**	0.41	0.21	0.17	0.43	0.82	0.11	0.09	0.18

**Table 6:** Comparison of explainable ML models in hydrogen yield prediction.

Models	Model Type	Hyper Parameters	Performance on Non-Linear Data	Generalizability	Explainable Ability	Industrial Suitability
**LR**	Linear	Low	Poor	prone to overfitting	Poor	Low
**KNN**	Instance-based	Low	Good	Good generalization with cross-validation	Excellent	High
**DTR**	Tree-based	Moderate	Moderate	Pruning improves generalization	Moderate	Moderate
**SVR**	Kernel-based	High	Excellent	Strong generalization with regularization	Excellent	High
**GBR**	Ensemble-based	High	Good	Good generalization with boosting	Good	High
**RFR**	Ensemble-based	Moderate	Moderate	Averaging improves generalization	Moderate	Moderate
**MLP**	Neural Network	High	Excellent	Dropout and early stopping improves generalization	Good	Moderate

## Data Availability

No new data were created or analyzed in this study.
